# Imaging findings of succinate dehydrogenase‐deficient renal cell carcinoma

**DOI:** 10.1002/ccr3.7799

**Published:** 2023-08-13

**Authors:** Wenqin Liu, Guiwu Chen, Jiaxin Meng, Xiaomin Liao, Yuhuan Xie

**Affiliations:** ^1^ Department of Ultrasound, Affiliated Dongguan Hospital, Southern Medical University Dongguan People's Hospital Dongguan China; ^2^ Department of Pathology, Affiliated Dongguan Hospital, Southern Medical University Dongguan People's Hospital Dongguan China

**Keywords:** computed tomography, magnetic resonance imaging, pathology, renal cell carcinoma, succinate dehydrogenase, ultrasonography

## Abstract

**Key Clinical Message:**

A 50‐year‐old man with a mass located in the left kidney was described by multimodal images, including ultrasonography, computed tomography, and magnetic resonance imaging. After surgical resection of the mass, pathological examination confirmed succinate dehydrogenase‐deficient renal cell carcinoma.

**Abstract:**

Succinate dehydrogenase‐deficient renal cell carcinoma (SDH‐deficient RCC) is a malignant tumor in the kidney associated with the loss of mitochondrial enzyme II. Due to its rarity, SDH‐deficient RCC is frequently misdiagnosed. We present multimodal imaging and pathologic findings in a 50‐year‐old male with SDH‐deficient RCC.

## Graphical abstract

Succinate dehydrogenase‐deficient renal cell carcinoma (SDH‐deficient RCC) is a malignant epithelial tumor that tends to occur in young males. Here, we present a case of SDH‐deficient RCC characterized by multimodal images and confirmed by pathological examination.

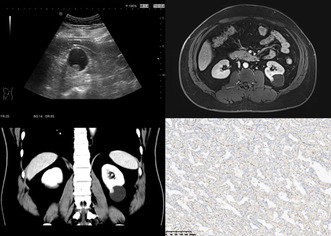

Succinate dehydrogenase (SDH)‐deficient renal cell carcinoma (RCC) is a malignant epithelial tumor defined by the absence of immunohistochemical expression of mitochondrial complex II. Due to its incidence accounting for only 0.05%–0.2% of all renal carcinomas, SDH‐deficient RCCs are frequently misdiagnosed.^1^ We report a case of SDH‐deficient RCC characterized by ultrasonography, computed tomography, and magnetic resonance imaging and confirmed by pathological examination.

A 50‐year‐old man with a history of hypertension, Hepatitis B, and smoking (30‐pack‐years) presented with a 1‐month of polypnea. Abdominal ultrasound revealed a cystic‐solid mass located in the left kidney (Figure 1). The mass was further characterized by computed tomography (Figure 2) and magnetic resonance imaging (Figure 3). The patient underwent surgical resection, and histology revealed a well‐circumscribed nested cells with flocculent cytoplasmic cysts containing eosinophilic material. Immunohistochemical staining performed with appropriate controls demonstrated that neoplastic cells expressed that pan‐cytokeratin, CK8/18, Vimentin, PAX‐8, GATA3, HNF1 Beta were positive; partial expression of AMACR, TFE‐3, E‐cadherin, and did not express SDHB CK7, CK20, RCC, CA, IX, CD10, CD117, or 34BetaE12. Ki‐67 was approximately 30%. Morphologic and immunohistochemical profiles were consistent with a diagnosis of SDH‐deficient RCC (Figure 4). No recurrence or metastasis was found during the 7‐month follow‐up postoperatively.

**FIGURE 1:**
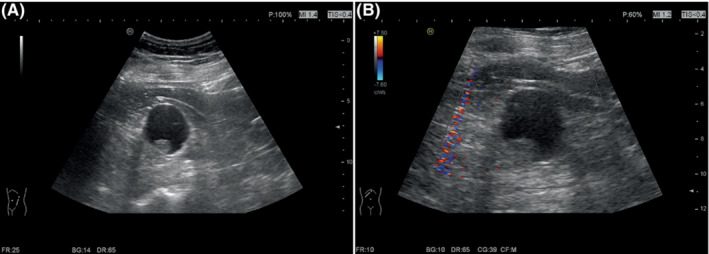
Ultrasonography of succinate dehydrogenase‐deficient renal cell carcinoma. (A) Grayscale ultrasound showed that the cystic‐solid mass was oval, well‐defined, and approximately 41 mm × 40 mm in size. (B) Color Doppler flow imaging showed that the solid component of the mass was isoechoic and approximately 16 mm × 9 mm in size without any blood flow signals.

**FIGURE 2:**
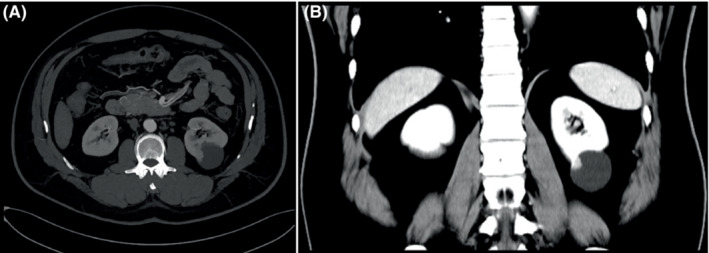
Computed tomography of succinate dehydrogenase‐deficient renal cell carcinoma. (A) Plain computed tomography showed that the cystic‐solid mass was low density and the solid component of the mass was approximately 37 HU. (B) Enhanced computed tomography showed that the solid component of the mass was obviously enhanced with 99 HU.

**FIGURE 3:**
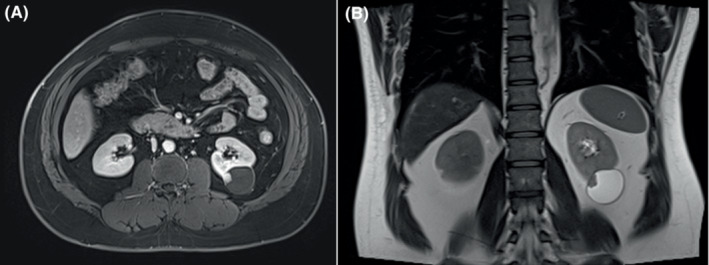
Magnetic resonance imaging of succinate dehydrogenase‐deficient renal cell carcinoma. (A) T1‐weighted imaging showed that the cystic‐solid mass had short signals and the solid component of the mass had long signals. (B) T2‐weighted imaging showed that the mass had long signals and the solid component of the mass had short signals.

**FIGURE 4:**
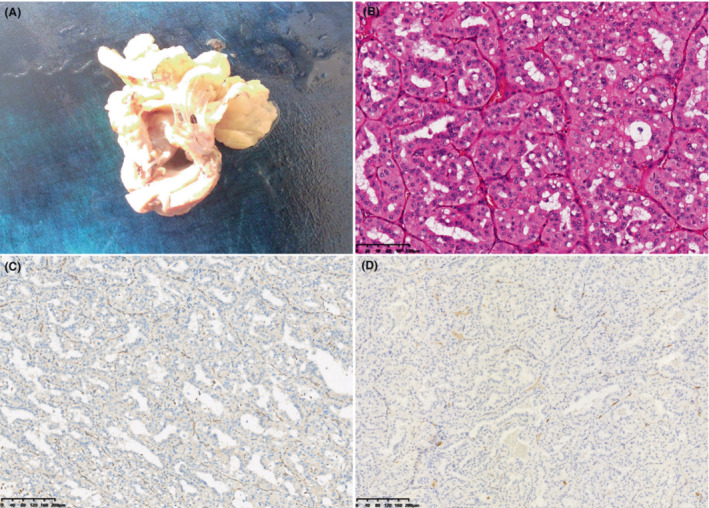
Pathology of succinate dehydrogenase‐deficient renal cell carcinoma. (A) The gross specimen showed that the cystic‐solid mass was grayish white and the solid component of the mass was papillary. (B) Hematoxylin–eosin staining showed the cells of the mass with abundant vessels presented in a nest or duct shape, the nucleus was enlarged and oval as well as the cytoplasm was eosinophilic and transparent. (C, D) Immunohistochemical staining showed SDHB and CD117 were negative.

SDH‐deficient RCCs tend to occur in relatively young adults with a mean age of 38 years with a slight male predominance (male‐to‐female ratio is 1.8). They can be unilateral or bilateral. They can be associated with paragangliomas, gastrointestinal stromal tumors, or pituitary adenomas.^2^ Abdominal ultrasound characteristically shows oval, well‐defined, and mix‐echoic with or without blood flow signals. Computed tomography and magnetic resonance imaging show that the masses could be cystic, cystic‐solid, or solid with obvious enhancement.^1^ Most SDH‐deficient RCCs have a favorable prognosis. Those with a low nuclear grade have lower metastatic risk, and patients can be treated with nephron‐sparing surgery. However, SDH‐deficient RCCs with unfavorable features, including coagulative necrosis, high nuclear grade, or sarcomatoid dedifferentiation have a higher metastatic rate and require radical nephrectomy.^3^

## AUTHOR CONTRIBUTIONS

**Wenqin Liu:** Data curation; writing – original draft. **Guiwu Chen:** Conceptualization; writing – review and editing. **Jiaxin Meng:** Investigation; validation. **Xiaomin Liao:** Investigation; validation. **Yuhuan Xie:** Resources; supervision.

## FUNDING INFORMATION

No funding was received for this study.

## CONFLICT OF INTEREST STATEMENT

The authors declare no conflicts of interest.

## TRANSPARENCY STATEMENT

We can confirm that this manuscript is an honest, accurate, and transparent account of the case being reported and that no important aspects of the case have been omitted.

## CONSENT STATEMENT

Written informed consent was obtained from the patient to publish this report in accordance with the journal's patient consent policy.

## Data Availability

The data used to support the findings of this study are available from the corresponding author upon request.
